# Prostate extracellular vesicles in patient plasma as a liquid biopsy platform for prostate cancer using nanoscale flow cytometry

**DOI:** 10.18632/oncotarget.6983

**Published:** 2016-01-22

**Authors:** Colleen N. Biggs, Khurram M. Siddiqui, Ali A. Al-Zahrani, Siddika Pardhan, Sabine I. Brett, Qiu Q. Guo, Jun Yang, Philipp Wolf, Nicholas E. Power, Paul N. Durfee, Connor D. MacMillan, Jason L. Townson, Jeffrey C. Brinker, Neil E. Fleshner, Jonathan I. Izawa, Ann F. Chambers, Joseph L. Chin, Hon S. Leong

**Affiliations:** ^1^ Department of Surgery, Western University, London, Canada; ^2^ Department of Oncology, Western University, London, Canada; ^3^ Translational Prostate Cancer Research Laboratory, Lawson Health Research Institute, London, Canada; ^4^ University Health Network, University of Toronto, Toronto, Canada; ^5^ Sandia National Laboratories, Albuquerque, NM, USA; ^6^ Department of Urology, University Medical Center Freiburg, Freiburg, Germany; ^7^ Department of Urology, University of Dammam, Dammam, Saudi Arabia; ^8^ Department of Mechanical and Materials Engineering, Western University, London, Canada

**Keywords:** prostate microparticles, extracellular vesicles, nanoscale flow cytometry, prostate cancer, liquid biopsy

## Abstract

**Background:**

Extracellular vesicles released by prostate cancer present in seminal fluid, urine, and blood may represent a non-invasive means to identify and prioritize patients with intermediate risk and high risk of prostate cancer. We hypothesize that enumeration of circulating prostate microparticles (PMPs), a type of extracellular vesicle (EV), can identify patients with Gleason Score≥4+4 prostate cancer (PCa) in a manner independent of PSA.

**Patients and Methods:**

Plasmas from healthy volunteers, benign prostatic hyperplasia patients, and PCa patients with various Gleason score patterns were analyzed for PMPs. We used nanoscale flow cytometry to enumerate PMPs which were defined as submicron events (100-1000nm) immunoreactive to anti-PSMA mAb when compared to isotype control labeled samples. Levels of PMPs (counts/μL of plasma) were also compared to CellSearch CTC Subclasses in various PCa metastatic disease subtypes (treatment naïve, castration resistant prostate cancer) and in serially collected plasma sets from patients undergoing radical prostatectomy.

**Results:**

PMP levels in plasma as enumerated by nanoscale flow cytometry are effective in distinguishing PCa patients with Gleason Score≥8 disease, a high-risk prognostic factor, from patients with Gleason Score≤7 PCa, which carries an intermediate risk of PCa recurrence. PMP levels were independent of PSA and significantly decreased after surgical resection of the prostate, demonstrating its prognostic potential for clinical follow-up. CTC subclasses did not decrease after prostatectomy and were not effective in distinguishing localized PCa patients from metastatic PCa patients.

**Conclusions:**

PMP enumeration was able to identify patients with Gleason Score ≥8 PCa but not patients with Gleason Score 4+3 PCa, but offers greater confidence than CTC counts in identifying patients with metastatic prostate cancer. CTC Subclass analysis was also not effective for post-prostatectomy follow up and for distinguishing metastatic PCa and localized PCa patients. Nanoscale flow cytometry of PMPs presents an emerging biomarker platform for various stages of prostate cancer.

## INTRODUCTION

A blood/urine based “liquid biopsy” for prostate cancer (PCa) could minimize the iatrogenic effects associated with needle core biopsies [1] and provide more accurate information regarding risk stratification. Prostate biopsies are needed because serum testing for Prostate Specific Antigen (PSA) is unable to distinguish patients with prostate cancer from patients with non-cancerous diseases of the prostate [2, 3]. Presently available “liquid biopsies” such as circulating tumor cells (CTCs) or fragments of tumor cell fragments (TCFs) as determined by the CellSearch Instrument are one such example, but these rely on epithelial markers that are not tissue specific [4]. CTCs are also rare in localized PCa disease [5], and therefore enumerating circulating TCFs by the CellSearch Instrument [4] or other high-throughput methods may be more appropriate for early stage disease.

Also known as prostate microparticles, these cell fragments taxonomically fall under the umbrella term, extracellular vesicles (EVs). The term “microparticle” is intended to represent entities that are 100-1000nm in diameter and released at the cell membrane whereas exosomes are EVs that are 40-100nm in diameter and are released by exocytotic mechanisms [6]. Microparticles have also been termed microvesicles, oncosomes, apoptotic bodies, ectosomes, prostasomes [7], with each of these terms providing etymologic clues regarding their origin and morphology. If released by the tumor, PMPs could conceivably express a subset of membrane-based biomarkers representative of the tumor's biology, whereas exosomes do not. Therefore, a high-throughput and multi-parametric method is needed to enumerate prostate microparticles and its associated biomarkers for correlation to clinical outcomes. Flow cytometry best fulfills this requirement as it is able to analyze 10^4^-10^5^ events per second at a multiple channel basis. However, conventional flow cytometry is not equipped to analyze submicron events because the optics and detection schemas are only designed to detect light scatter from events larger than 3 microns.

**Figure 1:**
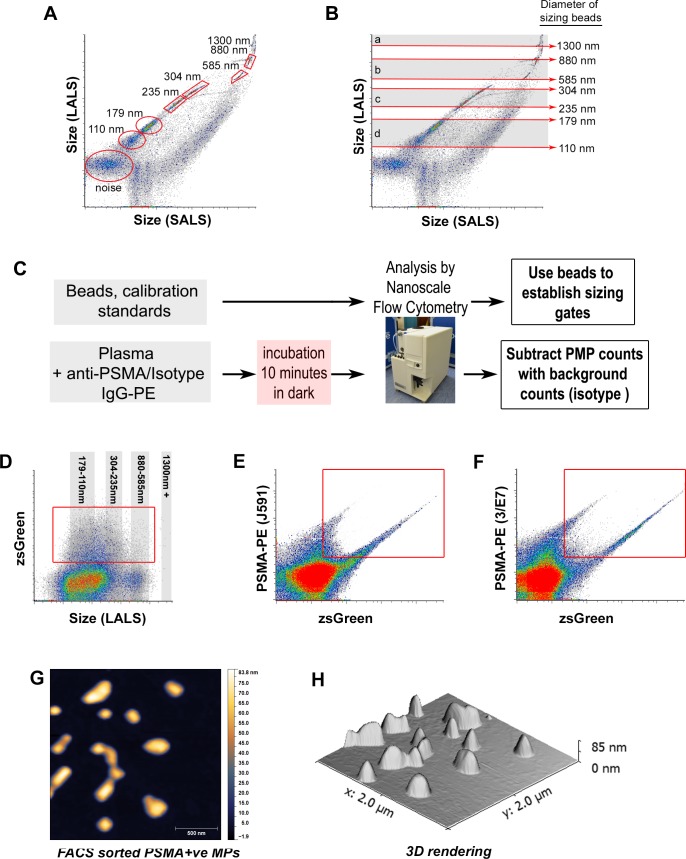
Nanoscale flow cytometry of various calibration sizing beads and *in vitro* generated prostate microparticles Analysis of silica beads of various diameters, 110nm, 179nm, 235nm, 304nm, 585nm, 880nm, 1300nm on the A50-Micro revealed several distinct populations **A.** and were used to define approximate size gates (shaded areas and red arrows) on the long angle light scatter (LALS) *vs*. short angle light scatter (SALS) scatterplot **B.**. **C.** Preparation of samples for nanoscale flow cytometric analysis is rapid and straightforward with minimal processing steps required. Microparticles (MPs) generated from PC3-zsGreen and LnCAP-zsGreen cultured cells were analyzed using nanoscale flow cytometry **D.**-**F.**, revealing dual positive MPs immunoreactive to J591 mAb (**E.**, Y-axis) and 3/E7 mAb (**F.**, Y-axis), and also contain zsGreen cytosolic protein (Y-axis in **D.**, X-axis in **E.**-**F.**). When sorted using FACS, individual vesicle-like structures < 500 nm in diameter were visualized using atomic force microscopy (**G.**, height channel), revealing a smooth ultrastructure **H.**.

Next generation instruments such as nanoscale flow cytometry are able to readily analyze events 100-1000nm in diameter using the same lasers and filters for analysis of multiple biomarkers on EVs such as PMPs in a high-throughput manner [8]. These improvements are largely due to enhanced optics designed to evaluate light scatter from submicron events and more sensitive photomultiplier tube (PMT) devices sensitive to fluorescence-based data generated by submicron entities [9]. No other instruments currently provide quantitative information of multiple biomarkers expressed on EVs. Therefore, nanoscale flow cytometry of EVs in plasma, serum, or urine [10] provides high-content information in a high-throughput manner. In this report, we describe its application with various sizing entities (calibration beads, liposomes), analysis of various sample types (cell culture media, plasma, serum), and the ability of nanoscale flow cytometry to enumerate PMPs defined as PSMA+ve events that exhibit a size range of 100-1000nm in diameter. Preparation of samples with probes for nanoscale flow cytometry is rapid and straightforward (Figure 1A). Applying this technology to retrospectively collected patient plasma samples with a median clinical follow-up of 3 years, we reveal that PMP levels are able to identify patients with Gleason Score 8+ disease from all other localized PCa patient cohorts, BPH patients, and healthy volunteers as well as patients with metastatic PCa disease.

## RESULTS

### Nanoscale flow cytometry of sizing standards

The A50-Micro nanoscale flow cytometer is capable of high-throughput and multi-parametric analysis of events between 100-1000nm, resolving various nanoparticles and calibration beads based on large angle light scatter (LALS) and small angle light scatter (SALS). The refractive index (RI) for silica is 1.43 and is similar to tissue (1.38 [13]) and silica nanoparticles of various sizes were used for establishing sizing gates along the LALS (Y axis) *vs*. SALS (X axis) scatterplot (Figure 1A-1B) instead of polystyrene microspheres (1.52). The nanoscale flow cytometer reveals 110nm, 179nm, 235nm, and 304nm silica nanoparticles as discrete subpopulations when analyzed together. Analysis of 100nm sized FITC-labeled liposomes (Figure S1A-C) and various latex beads (Figure S1D-E) were also readily detected by the A50-Micro nanoscale flow cytometer with conventional flow cytometry yielding non-linear results (Figure S1F).

### Suitability of PSMA antibodies for detection of *in vitro* generated prostate microparticles

To identify prostate microparticles in any liquid sample, anti-PSMA mAb and isotype controls are added to samples and incubated prior to analysis on the A50-Micro nanoscale flow cytometer (Figure 1C). The anti-PSMA mAb 3/E7 clone is specific for the cell membrane of LNCaP and BPH cells whereas the J591 mAb clone was only immunoreactive for LNCaP cells (S2). Microparticles generated i*n vitro* exhibited zsGreen fluorescence within a size range of 110-235nm (Figure 1D) and binded anti-PSMA clones J591-PE and 3/E7-PE mAbs (Figure 1E-1F). Dual-positive events were sorted by fluorescence activated cell sorting (FACS) onto mica coverslips for atomic force microscopy (AFM) imaging (Figure 1G-1H) [12], revealing a vesicle ultrastructure, exhibiting diameters of 100-250nm (Figure 1G).

### Enumeration of prostate microparticles in patient plasma samples and correlation to prostate specific antigen serum levels

**Figure 2:**
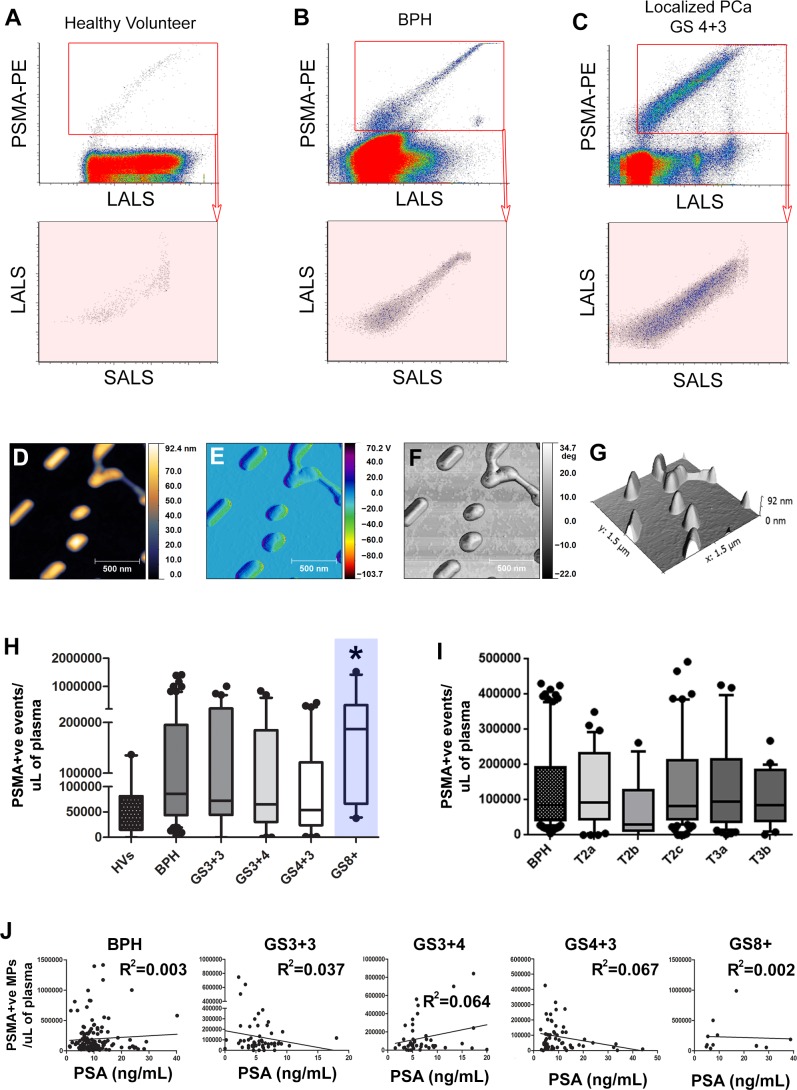
Prostate Microparticles (PMPs) are significantly elevated in prostate cancer patients with Gleason Score≥8 disease Representative PSMA *vs*. Size (LALS) scatterplots of various patient plasmas (healthy volunteer, BPH, localized PCa; **A.**-**C.**, left to right). PMPs were isolated by FACS for Atomic Force Microscopy (AFM) with the height channel **D.**, amplitude channel **E.**, phase channel **F.**, and 3D rendering **G.** of isolated PMPs. PMP levels are significantly higher in GS≥8 disease compared to the other GS patterns (**H.**, blue highlight = GS > 8) but no differences were observed between pathology tumor stages **I.**. Correlations of PMP *vs*. PSA serum levels for each Gleason Score pattern **J.**, revealed no statistically significant correlation. * denotes *p* < 0.05. *N* > 25 each group. One-way ANOVA.

Patient plasmas from healthy volunteers (*n* = 22, median age = 24 yrs, age range = 21-37 yrs, 13 male/9 female), patients diagnosed with benign prostatic hyperplasia (BPH, *n* = 156), localized prostate cancer (*n* = 256), and with castration resistant prostate cancer (*n* = 67) were analyzed and PSMA+ve submicron events were enumerated in each of these plasma samples (Figure S4). In Figure 2A-2C, representative PSMA *vs*. LALS scatterplots reveal the distribution and relative abundance of prostate microparticles in samples from healthy volunteers, BPH patients, and patients with Gleason Score 4+3 PCa respectively. AFM imaging was performed on PSMA+ve submicron events in patient plasma isolated by FACS to confirm their vesicular morphology (Figures 2D-2G and S3). There were significantly fewer prostate microparticles in plasmas from healthy volunteers compared to other patient plasma types (Figure 2H, far left bar, *denotes *p* < 0.05, one-way ANOVA). When comparing all Gleason Score patterns, only plasmas from patients with Gleason Score≥8 exhibited a significantly higher concentration of prostate microparticles compared to samples from patients with other Gleason Score patterns (Figure 2H, *denotes *p* < 0.05, one-way ANOVA). When TMN staging was considered, no significant differences were observed, indicating that the concentration of prostate microparticles in patient plasmas does not correlate to tumor stage (Figure 2I). No correlations were found between prostate microparticle counts and PSA levels regardless of the Gleason Score pattern evaluated (Figure 2J).

### Elevated levels of prostate microparticles in metastatic prostate cancer patients

**Figure 3:**
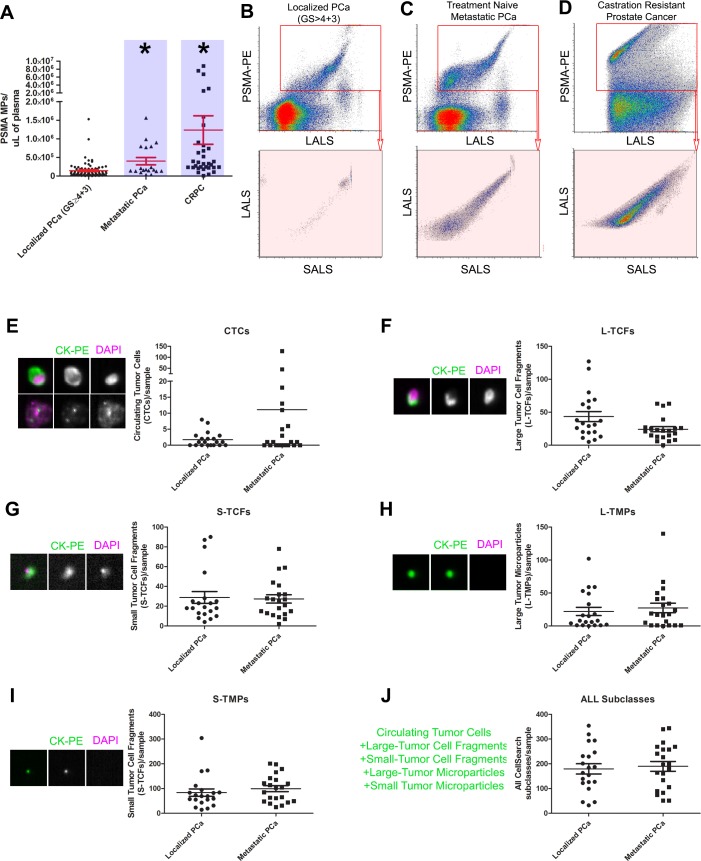
Nanoscale flow cytometry of prostate microparticles identifies patients with metastatic disease PSMA+ve microparticles, also known as PMPs, are significantly higher in patients with treatment naive metastatic prostate cancer (PCa) and castration resistant prostate cancer (CRPC) compared to patients with localized PCa (blue highlights, **A.**-**D**.). When compared to CellSearch Circulating Tumor Cell analysis, in which EpCAM+CK+CD45- events are enumerated by image-based flow cytometry, subclasses of CellSearch events were not able to distinguish localized PCa patients from metastatic PCa patients on the basis of: circulating tumor cells (CTCs, **E.**), Large Tumor Cell Fragments (L-TCFs, **F.**), Small Tumor Cell Fragments (S-TCFs, **G.**), Large Tumor Microparticles (L-TMPs, **H.**), Small Tumor Microparticles (S-TMPs, **I.**), and all CellSearch events combined **J.**. * denotes *p* < 0.05, *N* > 25 each group, one-way ANOVA.

CellSearch CTC and subclass analysis [14] and PMP analysis using nanoscale flow cytometry was performed on plasmas from treatment naive metastatic (prior to first-line therapy) and castration resistant prostate cancer (CRPC) patients (S5). PMP levels were significantly higher in CRPC and treatment naive metastatic PCa patient plasmas (Figure 3A-3D, *denotes *p < 0.05*, one-way ANOVA) when compared to those from localized PCa patients but did not exhibit significant correlation with serum PSA values (R^2^ = 0.42, *p > 0.17*). CellSearch subclass analysis was performed on a cohort of localized (N0,M0) and metastatic PCa patients (*n > 25* each) in which CTCs (Figure 3E), large tumor cell fragments (L-TCFs, Figure 3F), small tumor cell fragments (S-TCFs, Figure 3G), large tumor microparticles (L-TMPs, Figure 3H), small tumor microparticles (S-TMPs, Figure 3I) were analyzed. No statistically significant differences were noted in any of the subclasses, including cumulative subclass analysis (Figure 3J).

### Changes in prostate microparticle levels post-prostatectomy

**Figure 4:**
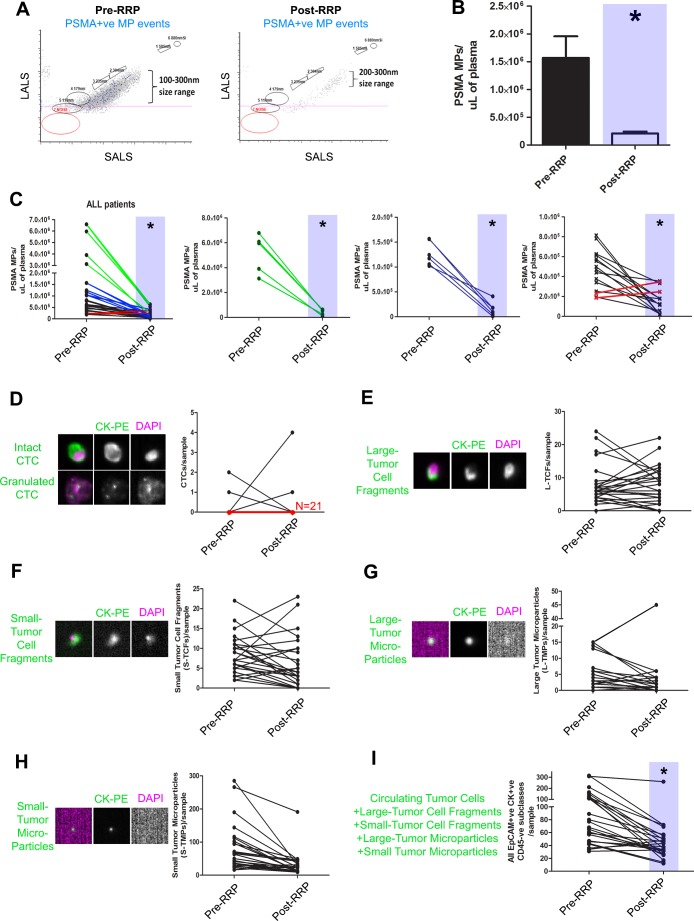
Decreases in prostate microparticle levels after prostatectomy as determined by nanoscale flow cytometry PMPs (PSMA+ve MP events) were enumerated in plasma samples collected pre- and 3 weeks post-prostatectomy (RRP) in PCa patients (*N* = 25). Decreases in PMPs were observed in the majority of patients post-RRP **A.** and was significant compared to pre-op PMP levels **B.**. Decreases in PMP levels varied between various patients (**C.**, left panel), and were stratified according to magnitude of decrease from pre-operative PMP levels (green, second left; blue, second from right; black and red, far right). Red lines indicate patients in which an increase in PMP levels was observed post-RRP. The same samples were submitted for CellSearch CTC analysis and all EpCAM+CK+CD45- subclasses were enumerated **D.**-**I.**, revealing no trend except when all CellSearch events are analyzed in aggregate **I.**. * denotes *p* < 0.05, *N* = 25 all groups, Paired *t*-tests, two-tailed.

**Figure 5:**
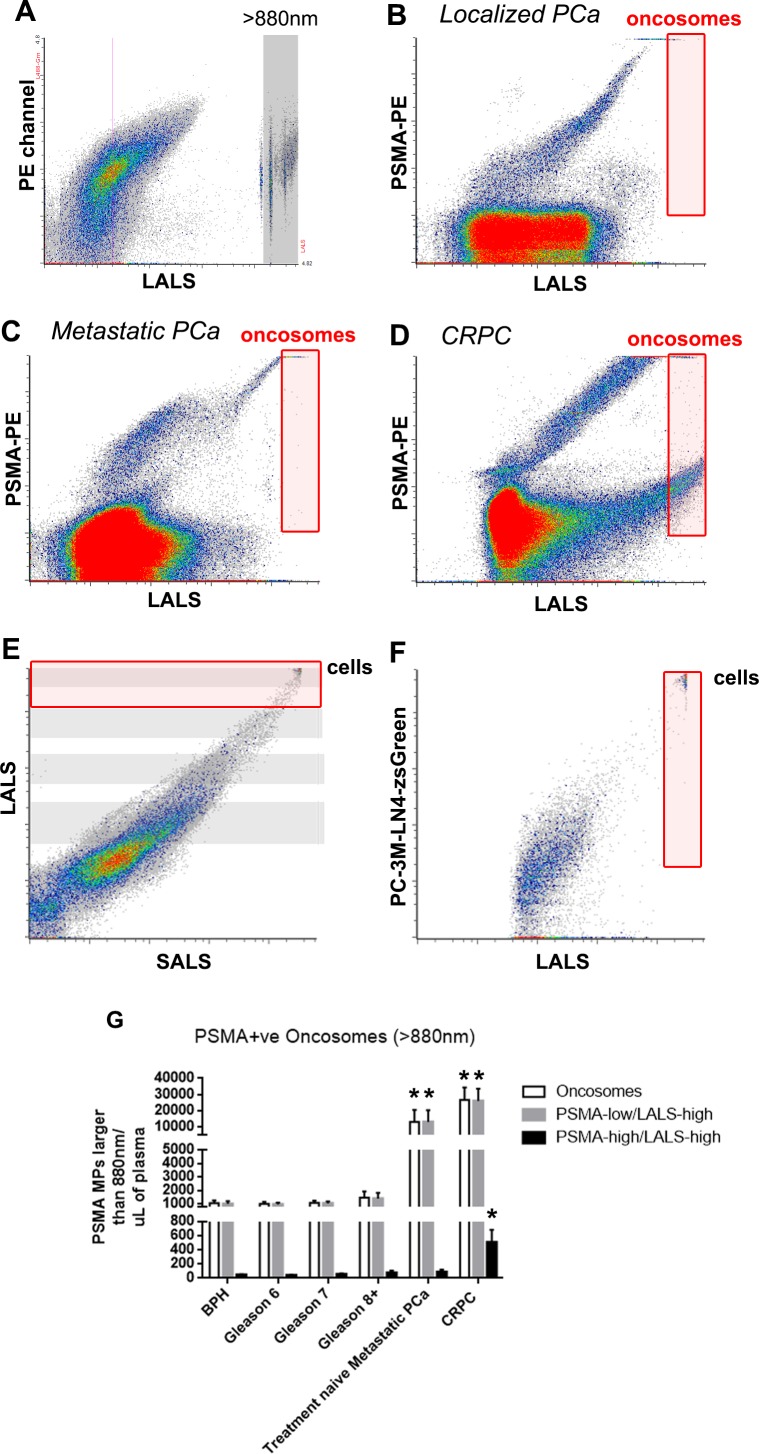
Prostate oncosome levels distinguish localized PCa patients from metastatic PCa patients To enumerate prostate oncosomes, PSMA+ve MPs that were larger than 880nm silica beads were used to set the analysis gate (red gate, **A.**-**D.**. Cells are detected in the upper corner of **E.** and **F.**). Metastatic PCa patient cohorts exhibited significantly higher prostate oncosome levels compared to localized PCa patient cohorts **G.**. Furthermore, PSMA-high/LALS-high events were significantly higher in the CRPC cohort compared to all other paired comparisons. One-way ANOVA, * denotes *p* < 0.05. *N* > 20 for each group.

Patients who underwent radical prostatectomy (RP) gave consent to have their blood analyzed pre-operatively and 3-weeks post-operatively to determine the change in PMP levels, CellSearch CTC Subclasses, and PSA after gland ablation (Figure S6). Figure 4A illustrates the change in PMP levels after RP (LALS *vs*. SALS scatterplot, PSMA+ve microparticles only gated, blue shaded areas denote groups, *T*-test *p < 0.05*). The median level of PMPs in postoperative patient samples was significantly lower than PMP levels from counterpart preoperative patient plasmas (Figure 4B). When paired for analysis (Figure 4C), the majority of patients exhibited significant decreases in PMP levels except for two patients in which PMP levels increased after RRP (red). Patients who exhibited > 90% decline in PMP levels (green, *n = 5*), and patients that exhibited between 90-70% decrease in PMP levels (blue, *n = 5*) also had undetectable PSA nadir levels (Figure S4). The remaining patient paired plasma samples that exhibited nomimal decreases in PMP levels (< 50%, black, *n = 10*), also exhibited undetectable PSA nadir levels. When CellSearch Subclass analysis was performed, downward trends in S-TCFs, L-TMPs, S-TMPs were observed but did not reach statistical significance (Figure 4E-4H), but when all CellSearch subclasses were cumulatively analyzed, a statistically significant downward trend was observed. Similarly, when oncosomes (PSMA+ve events larger than 1μm [15]) were enumerated, no differences were observed between all Gleason score patterns (red shaded gates, Figure 5). Only metastatic PCa patient plasmas exhibited significantly higher oncosomes counts relative to the localized PCa cohorts (*denotes *p < 0.05*, one-way ANOVA).

## DISCUSSION

We present a “liquid biopsy” for cancer diagnostics and clinical followup which relies on enumeration of prostate microparticles in patient plasmas by nanoscale flow cytometry. Enumeration of PMPs in plasma is highly dependent on monoclonal antibodies specific for PSMA and were highest in Gleason Score≥8 patient plasmas, but no correlation was observed with other Gleason Score patterns nor with pathological tumor stages. Plasmas from metastatic and castration resistant prostate cancer patients exhibited significantly higher prostate microparticle levels and oncosomes levels than those from localized prostate cancer patients.

The biomarker used in these studies was PSMA but is likely not prostate-specific as it has been shown to be expressed in other tissues (salivary glands, duodenum, kidney) and even in tumor vasculature of numerous solid tumors [16, 17]. PMPs were observed in a subpopulation of healthy volunteer (HV) female patient plasmas, but these levels were significantly lower than those present in localized PCa patient plasmas. PMP levels could also be used for prognostication following prostatectomy if they remain high despite a PSA undetectable nadir state. In the majority of patients who underwent prostatectomy, a significant drop in PMP levels was observed at three weeks post prostatectomy, and in some cases, negligible prostate microparticle counts were observed thus demonstrating the effectiveness of prostatectomy in decreasing tumor burden. However, there were minimal changes in prostate microparticle counts in a minority of patients, whereas PSA levels were at an undetectable nadir at three weeks post-prostatectomy. This could suggest either microscopic residual local disease or occult metastases or that local invasion has occurred in these men. PSMA as a prostate-specific marker may not be ideal, but the observation of low to negligible counts of PSMA+ve microparticles in healthy volunteers as well as the significant decrease in PSMA+ve microparticles in patients post-prostatectomy reveals its clinical potential as a first generation biomarker for prostate cancer microparticles.

Unanticipated were the high PMP counts observed within the BPH cohort; initially we hypothesized that BPH plasmas would not exhibit high PMP counts based on previous findings in urine [18]. These new findings reveal potential mechanisms of EV release into the blood circulation, which may have overlap with how PSA is released by the prostate into the blood, levels which were also elevated in the BPH cohort. An additional biomarker that is cancer specific (over-expressed across various tumor types) which is present on the surface of the microparticle would likely decrease the number of prostate cancer microparticle events in healthy volunteer or BPH patient plasmas. This would take advantage of the capabilities of nanoscale flow cytometry to perform enumeration of dual-biomarker or triple-biomarker positive microparticle events (100-1000nm) in a high-throughput manner with minimal consumption of patient plasma samples. Overall, enumeration of prostate microparticles or oncosomes could be used to improve population screening methods but will require many prospective studies to determine the AUC, specificity, and sensitivity of such a blood test.

## MATERIALS AND METHODS

### Patient sample preparation and ethics

Prostate cancer (PCa) patient plasmas or serum samples were attained through the Ontario Institute for Cancer Research Tumor Bank and the University Health Network Genitourinary BioBank (Toronto, ON) under Western University Research Ethics Board (REB) approved Ethics Applications # 103156 and 103409. Only samples from patients with minimum 3 years follow-up were included to avoid patients that upstaged/upgraded during that time.

Whole blood for CTC Enumeration by the CellSearch Instrument (Janssen Diagnostics Inc.) was collected into CellSave vacutainers (10mL volume, Janssen Diagnostics Inc.). To prepare plasma from whole blood for prostate microparticle analysis, whole blood was collected in K2-EDTA Vacutainers (BD Biosciences Inc.) and spun at 1500×g's for 10 minutes. The plasma layer was removed, aliquoted and then stored at −80°C. To prepare serum, whole blood was collected in BD Serum Vacutainers (BD Biosciences Inc.) and left overnight at room temperature. The tube was then spun at 1500×g's for 10 minutes and the serum layer was removed, aliquoted and stored at −80°C.

### Demonstrating the sizing resolution of the apogee A50-micro nanoscale flow cytometer

Three types of microspheres (silica, latex, liposomes) were analyzed using the A50-Micro Nanoscale Flow Cytometer (Apogee FlowSystems Inc.), the FACSCalibur III (Becton Dickinson Inc.) and dynamic light scattering instrumentation (ALV/CGS-3 Compact Goniometer). Silica beads were purchased from Apogee FlowSystems Inc.. Latex beads (100nm, 200nm, 500nm and 1μm Tetraspek beads; LifeTechnologies Inc.) were diluted 1:10000 prior to analysis on the A50-Micro Nanoscale Flow Cytometer. Liposomes were diluted 1:10000 prior to analysis on the A50-Micro. For synthesis of liposomes please refer to Supplementary Methods.

### Characterization of PSMA expression on microparticles generated from cell lines

LNCaP and PC3 cells (ATCC Inc.) were infected with lentivirus encoding cytoplasmic zsGreen protein (pLVX-zsGreen, Clontech Inc.). Cell pellets were resuspended in ddH_2_O and left for one hour at room temperature in the dark. The cells were spun down again at 1500×g's for 5 minutes and the supernatant was reserved, aliquoted, and stored at −80°C. To detect prostate microparticles, 1μL of anti-PSMA mouse IgG_2b_-PE (3/E7 clone, J591 clone, each 408.42μg/mL, mAb purification according to [11]) was added to 20μL of suspended microparticles and incubated at room temperature for 10 minutes in the dark. Samples were then diluted in 600uL of ddH_2_O and analyzed on the A50-Micro.

### Staining of microparticles and analysis by nanoscale flow cytometry and atomic force microscopy

To detect prostate microparticles in patient plasma samples, anti-PSMA mouse IgG_2b_-PE (clone 3/E7) was added to 20μL of patient plasma. Negative isotype stained controls consisted of mouse isotype control IgG_2b_-PE (Beckman Coutler Inc.) added to 20μL of patient plasma. Gates for each microparticle population were established by analyzing the isotype control first, modifying the gains for each PMT as necessary, and then analyzing the antibody labeled samples.

For microparticles generated *in vitro*, these were isolated by FACS as previously described in [12], in which PSMA+ve and zsGreen+ve co-expressing microparticle events were sorted onto cleaved mica coverslips (Ted Pella Inc. Redding, CA) to isolate prostate microparticles whilst eliminating non-target EVs and serum protein. After sorting 1000-3000 events onto the mica coverslips, samples were allowed to dry in 50 mL Falcon tubes for 5 minutes at 70°C before imaging with atomic force microscopy (AFM). No washing of the samples was performed. Images were acquired with tapping mode AFM (Dimension V, Veeco Inc.). Rectangle cantilevers with nominal spring constant of 40 N/m and tip radius of < 10 nm were used to acquire high resolution topographic images. All measurements were done at low driving force to avoid damaging samples.

### Circulating tumor cell (CTC) and EpCAM+CK+CD45- cellSearch subclass analysis in patient whole blood samples

Whole blood from patients at pre- and post-radical retropubic prostatectomy (3 weeks post) were collected into CellSave Vacutainers and then submitted to CellSearch Analysis. CTC counts for each blood sample were determined by two trained viewers and EpCAM+ve CK+ve CD45-ve CellSearch Subclasses were enumerated by an independent viewer relying on parameters established in [4].

### Statistical analysis of flow cytometric data

All flow cytometry data was collected using the A50-Micro instrument acquisition software and all data was analyzed using GraphPad Prism software. In all analyses, one-way ANOVA (Bonferroni Correction applied) was used to evaluate statistical significance across the different groups and subgroups of patient plasmas. To correlate PSMA+ve MPs to PSA blood levels, linear regression analysis was used.

## SUPPLEMENTARY FIGURES AND TABLES



## References

[1] Loeb S, Carter HB, Berndt SI (2011). Complications after prostate biopsy: data from SEER-Medicare. J. Urol.

[2] U.S. Preventive Services Task Force - Screening for Prostate Cancer Current Recommendation http://www.uspreventiveservicestaskforce.org/prostatecancerscreening/prcascres.pdf.

[3] Catalona WJ, Smith DS, Ratliff TL (1991). Measurement of prostate-specific antigen in serum as a screening test for prostate cancer. N. Engl. J. Med..

[4] Coumans FAW, Doggen CJM, Attard G (2010). All circulating EpCAM+CK+CD45- objects predict overall survival in castration-resistant prostate cancer. Ann. Oncol.

[5] Lowes LE, Lock M, Rodrigues G (2012). Circulating tumour cells in prostate cancer patients receiving salvage radiotherapy. Clin. Transl. Oncol.

[6] Duijvesz D, Luider T, Bangma CH, Jenster G (2011). Exosomes as biomarker treasure chests for prostate cancer. Eur. Urol.

[7] Ronquist GK, Larsson A, Stavreus-Evers A, Ronquist G (2012). Prostasomes are heterogeneous regarding size and appearance but affiliated to one DNA-containing exosome family. Prostate.

[8] Van der Pol E, Coumans FAW, Grootemaat AE (2014). Particle size distribution of exosomes and microvesicles determined by transmission electron microscopy, flow cytometry, nanoparticle tracking analysis, and resistive pulse sensing. J. Thromb. Haemost.

[9] Chandler WL, Yeung W, Tait JF (2011). A new microparticle size calibration standard for use in measuring smaller microparticles using a new flow cytometer. J. Thromb. Haemost.

[10] Drake RR, Kislinger T (2014). The proteomics of prostate cancer exosomes. Expert Rev. Proteomics.

[11] Wolf P, Freudenberg N, Bühler P (2010). Three conformational antibodies specific for different PSMA epitopes are promising diagnostic and therapeutic tools for prostate cancer. Prostate.

[12] Leong HS, Podor TJ, Manocha B, Lewis JD (2011). Validation of flow cytometric detection of platelet microparticles and liposomes by atomic force microscopy. J. Thromb. Haemost.

[13] Dirckx JJJ, Kuypers LC, Decraemer WF (2005). Refractive index of tissue measured with confocal microscopy. J. Biomed. Opt.

[14] Coumans FAW, Doggen CJM, Attard G (2010). All circulating EpCAM+CK+CD45- objects predict overall survival in castration-resistant prostate cancer. Ann. Oncol.

[15] Di Vizio D, Morello M, Dudley AC (2012). Large oncosomes in human prostate cancer tissues and in the circulation of mice with metastatic disease. Am. J. Pathol.

[16] Chang SS, Reuter VE, Heston WD, Gaudin PB (2001). Metastatic renal cell carcinoma neovasculature expresses prostate-specific membrane antigen. Urology.

[17] Haffner MC, Laimer J, Chaux A (2012). High expression of prostate-specific membrane antigen in the tumor-associated neo-vasculature is associated with worse prognosis in squamous cell carcinoma of the oral cavity. Mod. Pathol.

[18] Işın M, Uysaler E, Özgür E (2015). Exosomal lncRNA-p21 levels may help to distinguish prostate cancer from benign disease. Front. Genet.

