# Supplementary material for: Prostate extracellular vesicles in patient plasma as a liquid biopsy platform for prostate cancer using nanoscale flow cytometry
Source: Oncotarget. 2016 Jan 22;7(8):8839–49. doi: 10.18632/oncotarget.6983 (PMC4891008; doi:10.18632/oncotarget.6983)
Supplement: Supplementary file 1 [file oncotarget-07-8839-s001.pdf]

Prostate extracellular vesicles in patient plasma as a liquid biopsy platform for prostate cancer using nanoscale flow cytometry

Supplementary Materials

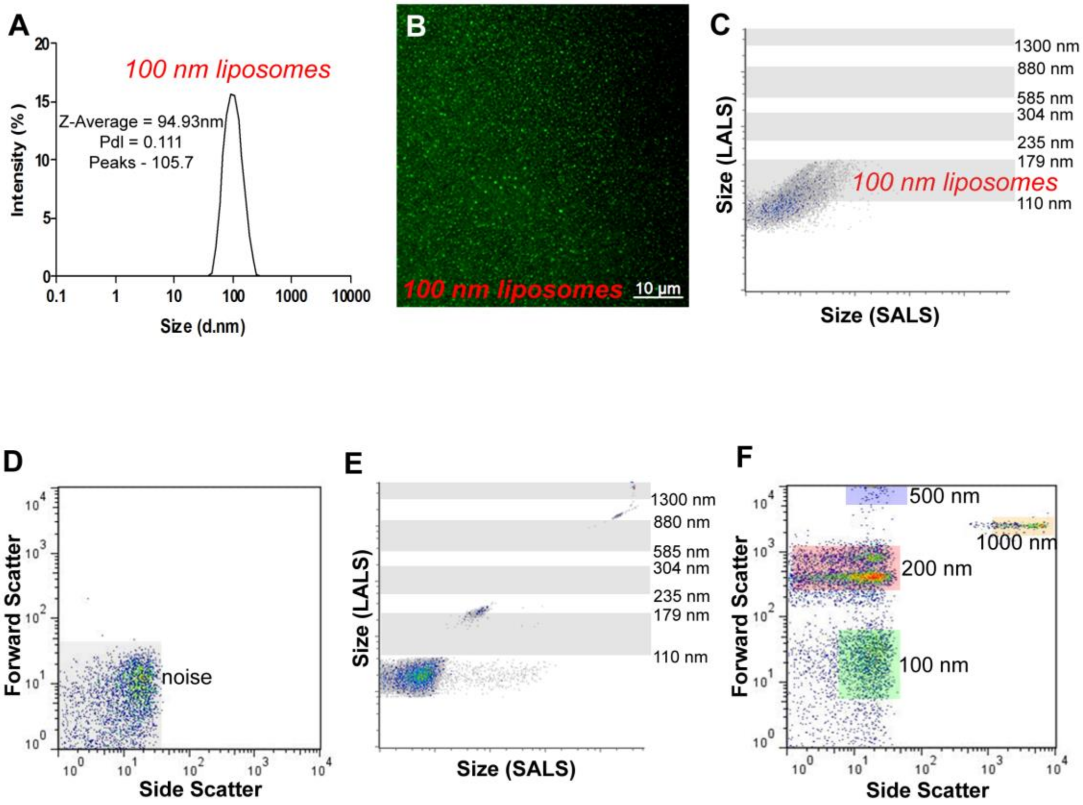

S1 – Supplementary Figure 1. Nanoscale flow cytometry of liposomes and latex beads.

A-C) FITC-labeled liposomes of a known diameter (100 nm) were analyzed by dynamic light scattering instrumentation (A), then on the A50-Micro nanoscale flow cytometer (C), revealing a population at the 110 nm size gate. FITC labelled liposomes are visible under widefield fluorescence microscopy (B) but not detectable when analyzed by conventional flow cytometry FACSCalibur III (D). Latex beads were not observed when using the FACSCalibur III. E) When latex beads of diameters 100nm, 200nm, 500nm and 1000nm were analyzed on the A50-Micro, specific populations could be distinguished on both the A50-Micro (E) and FACSCailibur III except for the 100nm bead size (F).

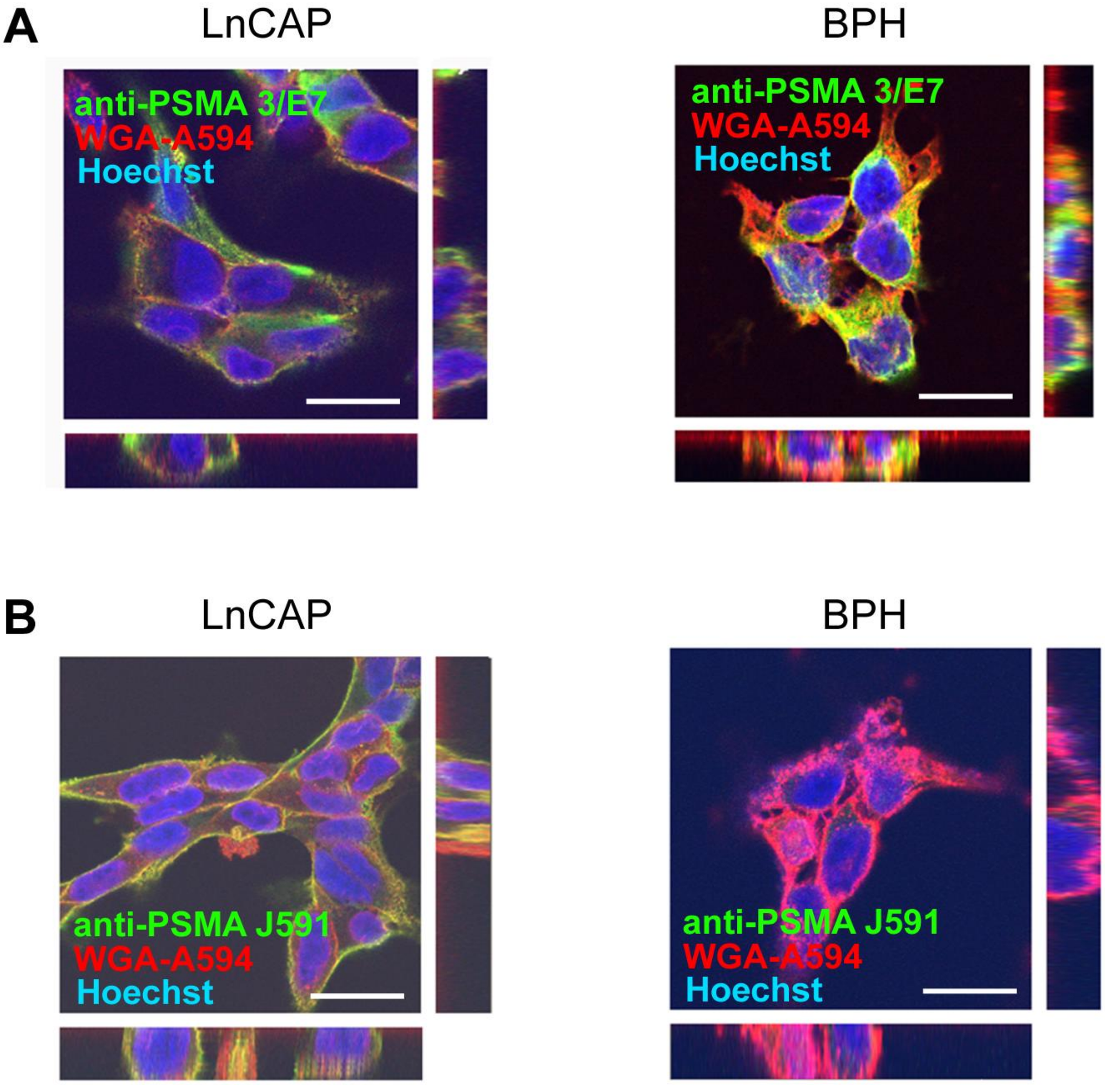

S2 – Supplementary Figure 2. Characterization of PSMA antibodies on cell lines. PSMA clone 3/E7 was used to fluorescently stain LnCAP cells (A, left panel) and BPH cells (right panel). PSMA mAb clone J591 was used to fluorescently stain LnCAP cells (B, left panel) BPH cells (right panel).

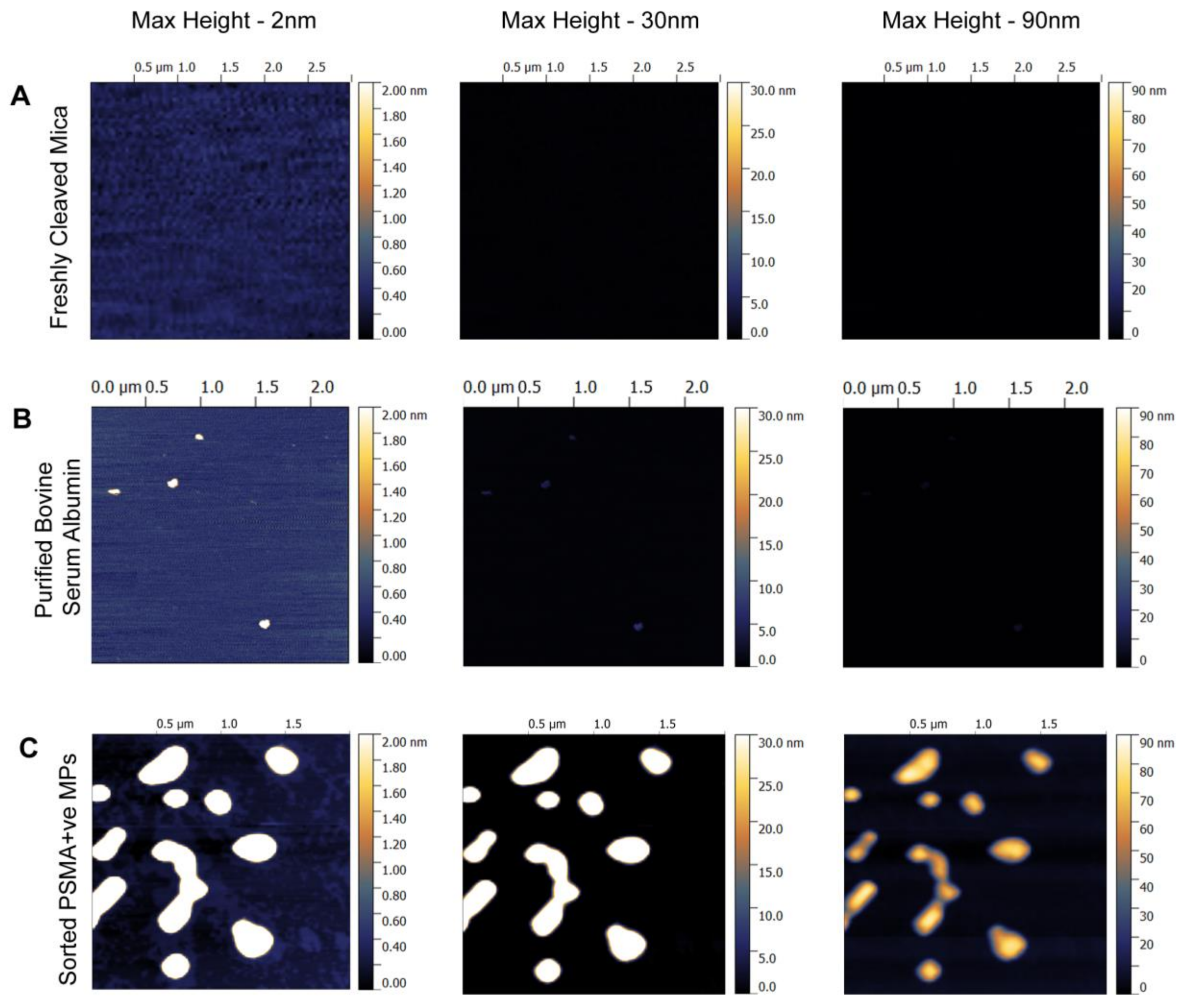

S3 – Supplementary Figure 3. Comparison of Atomic Force Microscopy (AFM) Images of Mica, Albumin, and Sorted PSMA+ve Microparticles. Height channel of AFM images of freshly cleaved mica with a 2nm, 30nm, and 90nm maximum LUT (A). (B) Height channel of AFM images of purified bovine albumin (10 ng/mL dilution) with the same LUT. (C) Height channel of AFM images of sorted PSMA+ve microparticles (10 ng/mL dilution) with the same LUT. Images in (C) correspond to Figure 1G-H.

S4 – Supplementary Table 1. Clinical Information for all PCa Patient Cohorts. The first set lists clinical information for all patients enrolled in data represented in Figure 2. The second set lists clinical information for all patients enrolled for research represented in Figure 3. The third set lists clinical information for all patients enrolled for research represented in Figures 4-5.

	Localized Prostate Cancer (N=265)	Benign Prostatic Hyperplasia (N=156)
Age<br>• 40-64 years<br>• 65-79 years	 • 170 (65%)<br>• 95 (35%)	 • 92 (59%)<br>• 64 (41%)
Mean PSA (±SEM, ng/mL)	13.88±62.22 (1.5 - 541)	10.07±5.82 (0.9 - 149)
Median PSA (ng/mL)	7.0	8.7
Median gland size (g)		67.5
pT Stage<br>• pT2a<br>• pT2b<br>• pT2c<br>• pT3a<br>• pT3b<br>• pTx	 • 38 (14.3%)<br>• 21 (7.9%)<br>• 75 (28.3%)<br>• 72 (27.2%)<br>• 34 (12.8%)<br>• 25 (9.4%)	
Gleason Score<br>• 6<br>• 7<br>○ 3+4<br>○ 4+3<br>• 8<br>• 9<br>• Unknown	 • 61 (23.0%)<br>• 113 (42.6%)<br>○ 53 (20.0%)<br>○ 60 (22.6%)<br>• 26 (9.8%)<br>• 15 (5.6%)<br>• 50 (18.9%)	

S5 – Supplementary Table 2. Clinical Information for Localized and Metastatic PCa Patient Cohorts Submitted to CellSearch CTC Analysis.

	Localized PCa (N=23)	Metastatic PCa (N=24)
Age (yrs±SD)	63.1±5.5	62.2±5.7
Pre-Op PSA (ng/mL ±SD)	7.83±3.6	
PSA Pre-first line (ng/mL ±SD)		35.0±49.9
pT Stage at Pre-Op<br>• pT1c<br>• pT2a<br>• pT2b<br>• pT2c<br>• pT3a	 • 13 (56%)<br>• 2 (9%)<br>• 2 (9%)<br>• 3 (13%)<br>• 3 (13%)	
Definitive Therapy<br>• External Beam Radiation Therapy<br>• Radical Retropubic Prostatectomy		 • 13 (54%)<br>• 11 (46%)
Adjuvant Radiation Therapy		19 (79%)
Adjuvant Hormone Therapy		19 (79%)
Bone Scan Positivity		9 (38%)
Biochemical Failure		24 (100%)

S6 – Supplementary Table 3. Clinical Information for Pre- and Post-RRP PCa Patient Cohort

	All Patients (N=25)	Significant Decrease in PCMPs (N=16)	Insignificant Decrease in PCMPs (N=9)
Age	61.05±6.4	60.6±6.4	64.6±7.6
Pre-Op PSA (ng/dL)	6.21±3.19	6.3±3.0	5.5±2.1
Prostate Volume (mL)	48.19±23.9	46.06±24.9	64.6±7.6
Biopsy Gleason Score<br>• 6<br>• 7<br>• ≥8	 • 11 (44%)<br>• 11 (44%)<br>• 3 (12%)	 • 5 (31.3%)<br>• 8 (50.0%)<br>• 3 (18.7%)	 • 6 (66.7%)<br>• 3 (33.3%)<br>• 0
# of Positive Biopsy Needle Cores	3.02±1.95	3.4±2.0	2.8±1.5
Tumor Greatest Dimension (mm)	16.26±8.2	19.25±7.7	14.6±6.7

Supplementary Methods:

Cell line Immunostaining - LNCaP, and BPH-1 cells (ATCC Inc.) were fixed with 2.5% paraformaldehyde (in PBS) and stained using a standard immunofluorescence staining protocol. Each cell line was stained separately with anti-PSMA mouse IgG_{2b} (clone 3/E7), anti-PSMA mouse IgG_1 (clone J591, as purified based on[9]), or a mouse IgG isotype control. A goat anti-mouse IgG-Alexa488 secondary antibody was used followed by staining with Wheat Germ Agglutinin-Alexa594 and Hoechst 33345 dye to stain the surface membrane and nucleus of stained cells (LifeTechnologies Inc.).

Liposome Synthesis and Characterization - these materials were used: L-α-phosphatidylcholine (Egg PC), 25-[N-[(7-nitro-2-1,3-benzoxadiazol-4-yl)methyl]amino]-27-norcholesterol (NBD-chol), 2-distearoyl-sn-glycero-3-phosphocholine (DSPC), 1,2-distearoyl-sn-glycero-3-phosphoethanolamine-N-[methoxy(polyethylene glycol)-2000] (DSPE-PEG), 1,2-dioleoyl-3-trimethylammonium-propane (DOTAP), Dimethyldioctadecylammonium (Bromide Salt) (DDAB), phospholipids and cholesterol (ovine wool, >98%) were purchased from Avanti Polar Lipids (Birmingham, AL). 1X phosphate buffered saline (PBS) was purchased from Gibco (Logan, UT). Lipids and cholesterol (chol) ordered from Avanti Polar Lipids were presolubilized in chloroform and were stored at -20°C. Liposome formulations include Egg PC:chol:NBD-chol (molar ratio % 65:33:2), Egg PC:Chol:DSPE-PEG:DOTAP:NBD-chol (60:32:3:3:2), Egg PC:chol:DDAB:NBD-chol (65:30:3:2), and DSPC:chol:DSPE-PEG:NBD-chol (54:42:2:2). Liposome components were mixed in solvent, and then dried under high vacuum to remove residual solvent. Dried lipid films were hydrated in 0.5X PBS at a concentration of 5 mg/ml then briefly bath sonicated. Finally, liposomes were extruded through either 1.0 µm, 0.4 µm or 0.05 µm polycarbonate filter membrane (minimum 21 passes).
